# Public Pension, Labor Force Participation, and Depressive Symptoms across Gender among Older Adults in Rural China: A Moderated Mediation Analysis

**DOI:** 10.3390/ijerph17093193

**Published:** 2020-05-04

**Authors:** Xin Gao, Tieying Feng

**Affiliations:** School of Public Policy and Administration, Xi’an Jiaotong University, Xi’an 710049, China; gaoxin1017@stu.xjtu.edu.cn

**Keywords:** public pension, new rural pension scheme, labor force participation, depressive symptoms, gender

## Abstract

Due to insufficient financial support and unceasing work, the rural elderly in China experience a range of mental disorders, and the most common one is depression. This study aims to investigate the association between public pension, labor force participation (LFP), and depressive symptoms for older men and women in rural China. A moderated mediation analysis is conducted using data in the 2015 wave extracted from the China Health and Retirement Longitudinal Study (CHARLS), a continuous national social survey. A total of 2709 available surveys were obtained in our analysis. Using PROCESS, results revealed that the income from China’s New Rural Pension Scheme (NRPS) was directly negatively related to depressive symptoms. However, LFP did not mediate the link between pension income (PI) and depressive symptoms in the total study population. The results of moderated mediation estimates indicated that gender significantly moderated the relationship between LFP and depressive symptoms. Specifically, for older women, the indirect effect of PI on depressive symptoms via LFP was significant, but not for the opposite sex. In order to improve the mental health of older adults in rural China, the policy makers and mental health therapists need to pay attention to the aforementioned factors.

## 1. Introduction

Depression is one of the most common mental disorders among older adults, and correlates with morbidity and suicide [1,2,3,4]. According to the World Health Organization, approximately 15% of adults aged 60 and over suffered from a mental disorder [5], and an estimated 76–85% of people suffering from mental disorders lacked access to treatment in developing countries [6]. Although no accurate depressed geriatric population calculations were made for China, about 40% of older adults had reported depressive symptoms according to the China Health and Retirement Longitudinal Study [7]. Additionally, the prevalence of depression in rural China is more serious than that in urban China [8,9]. This difference is particularly relevant to insufficient social support [10] and unceasing work in rural areas.

The New Rural Pension Scheme (NRPS) launched in late 2009 is an important approach to promoting the quality of life in rural China. As the largest national transfer program, NRPS had covered all rural areas in China by the end of 2012, even though the amount of pension was still meager. Indeed, the social security from NRPS could not fully meet the needs of rural older adults and ease the growing financial burden of old age [11]. Thus, the majority of the geriatric population in rural China still has to work all the way throughout old age until they are no longer physically capable, which deserves special attention. Previous studies have suggested that late-life labor force participation (LFP) is negatively associated with old-age benefits [12,13] and positively associated with depressive symptoms [14]. Namely, LFP may serve as an important missing link between pension income (PI) and depressive symptoms among the rural elderly. In addition, part of these associations reflected gender and socioeconomic disparities [15]. While previous literature found a strong income–mental health gradient among older adults in the context of developed countries [16,17], no study has yet considered both the mediating role of LFP and the moderating role of gender in the correlation between PI and depressive symptoms among geriatric population in developing regions. In this study, we investigated the direct and indirect associations between PI, LFP, and depressive symptoms in rural China. Additionally, we determined whether gender moderated the relationship between LFP and depressive symptoms. Our study would contribute to the development of relevant and targeted depression prevention for the vulnerable older population.

### 1.1. Public Pension and Depression

Public pension is an important variable for understanding geriatric depression. Older adults are more likely to be exposed to prolonged poverty spells than other groups, which is a potential risk of depressive symptoms [18,19]. The situation would be worse with regard to the rural elderly because of limited income sources. As an important formal social support, the income from NRPS thus plays an important role in their mental health with two possible explanations. On the one hand, financial gains over a short period of time may lead to more detectable improvements in mental health compared with physical health [20,21]. For example, empirical studies on NRPS found that even a relatively modest pension may lead to better mental health of the rural older adults. However, no significant effect of PI on self-reported physical health was found in research [22]. On the other hand, PI could improve mental health by promoting the quality of life among older adults. A comparative study from South Africa and Brazil indicated that national pension had a substantial impact on the prevalence and depth of poverty and the level of life satisfaction [23]. These findings provide important insights into the impact of public pension on geriatric depression, particularly in low- and middle-income countries.

### 1.2. Public Pension, LFP, and Depression

In view of rapid ageing and deteriorating economic conditions, LFP may have an impact on the link between public pension and depression among older adults. Although promoting LFP of the elderly has long been endorsed to deal with population ageing in many developed regions [24], there is so far little consensus on the outcome of LFP on mental health among older adults. For example, studies in rural China and Lebanon underscored the importance of paid work on maintaining a sense of meaning and income, and revealed that paid work had a protective effect on depression [8,25]. However, an analysis on six developing countries showed a significant negative relationship between LFP and depressive symptoms [14]. A reduction in leisure time and social activities led by LFP could be the path of this association [26]. It has been shown that fewer social connections and less participation in social activities predict a higher risk of cognitive decline and mental illness among the elderly [27,28,29]. For example, a fixed-effects analysis in China indicated that rural residents took part in fewer social activities and had a significantly higher risk of depression than their urban counterparts [30].

The relationship between public pension and LFP has also been well documented in recent decades [31,32]. Individual wealth carries weight in the decision whether to retire or continue to work. For instance, wealthier people tend to retire earlier [33]. PI can provide a financial incentive for older people to retire [34]. In fact, even though theory may make a clear prediction of the relationship between PI and LFP in older adulthood, it has not always been supported by empirical evidence. For example, Louise Grogan and Fraser Summerfield suggested that PI increased the likelihood that individuals considered themselves pensioners and thus had negative attitudes to ageing and reduced the labor supply in paid work [35]. However, Christopher J. Ruhm argued that old-age pension was likely to reduce labor supply at some ages but to increase it at others, resulting in a more complicated and ambiguous aggregate impact than was frequently realized [31]. Manxiu Ning and colleagues found no evidence that the income from NRPS significantly reduced labor supply among the elderly [15]. To date, however, no study has consistently noted the association between public pension and LFP. Furthermore, the role of LFP as a potential mediator in the link between public pension and depressive symptoms has not been examined.

### 1.3. Consideration of Gender

When exploring the mediating effect of LFP on depressive symptoms among older adults, it is important to consider how this effect varies across gender for several reasons. First, according to the traditional gender division of labor, women are always engaged in a greater number of household affairs [36], while men are more involved in productive or paid work. Take Brazil for instance, the proportion of older males that were employed was 41% higher than that of females [37]. Second, with cardiorespiratory fitness and musculoskeletal capacity [38], older men often have quite different work patterns compared to older women [36]. As a result, they are more likely to be exposed to a stressful work environment, and be at a higher risk of onset and accumulation of illness, which in turn may increase the likelihood of developing depressive disorders [39]. Third, unlike men, inter-role conflict is a major work-related risk for women, because they still have to perform the majority of household tasks when they participate in work. The more roles the older women play in daily life the more pressure they have to cope with, which is commonly associated with a bad mood and reduced life satisfaction [40]. Fourth, late-life depression has been shown to be gender related. Luppa et al. (2016) reported that the depression prevalence ratio of men to women was 1:1.4–2.2 [41]. More recently, one empirical study based on nationally representative data from the Health and Retirement Study (HRS) also suggested that compared to men, women exhibited higher depressive symptoms from ages 51 to 85. The ability to report feelings of depressed mood is an important cause of this difference [42]. Although previous studies have reported significant gender differences in LFP and depressive symptoms, no study to date has considered the gender differences when investigating the mediating effect of LFP on the link between PI and depressive symptoms.

### 1.4. Current Study

**Figure 1:**
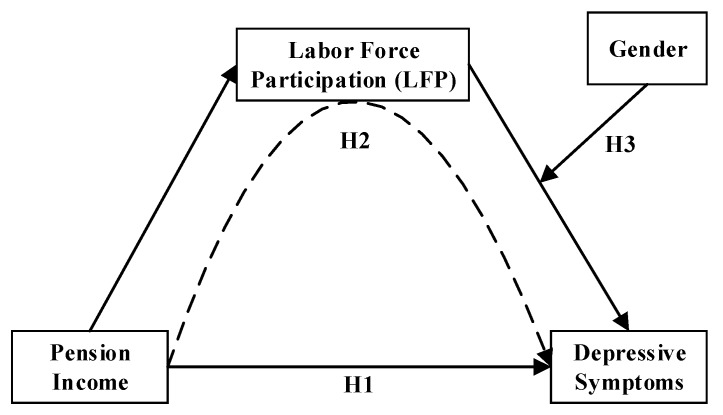
Conceptual diagram of the moderated mediation analysis. Note: H1—Hypothesis 1; H2—Hypothesis 2; H3—Hypothesis 3.

Previous studies have investigated the potential associations between public pension, LFP, and depressive symptoms. To date, however, no study has combined these three variables together in one model, and little has considered how these relationships differ across gender. The current study makes three contributions to better clarify the relationships between public pension, LFP, and depressive symptoms across gender in rural China. First, as in the research by Xi Chen and colleagues [21], we hypothesized that older adults with higher PI would experience fewer depressive symptoms (see in Figure 1, H1), and investigated this assumption in the study population. Second, we addressed the mediating effect of LFP on the relationship between PI and depressive symptoms, and assumed that an increase in PI would be associated with a decrease in LFP [43], which in turn would be associated with a decline in depressive symptoms in the overall sample (see in Figure 1, H2). Third, considering the gender differences of LFP and depressive symptoms, we assumed that the impact of LFP on depressive symptoms would vary across gender (see in Figure 1, H3).

## 2. Materials and Methods 

### 2.1. Data Source and Study Population

The data discussed in this study came from the China Health and Retirement Longitudinal Study (CHARLS), a continuous national social survey project conducted by Peking University. The baseline survey was officially launched in 2011. The aim of CHARLS is to provide various and quality data on the ageing process and the quality of life of participants aged 45 and over. Participants in the 2015 wave were recruited through a multistage, stratified sampling procedure. This study focused on the connections between PI, LFP, and depressive symptoms in older adults. As such, we excluded respondents under the age of 60, respondents who were not agricultural household registration (‘Hukou’), and respondents who had missing values for some key analysis variables. Finally, a total of 2709 available surveys were obtained in our analysis.

### 2.2. Measurements

#### 2.2.1. Depressive Symptoms

The CES-D-10, a short form (10-item) tool from the Center for Epidemiologic Studies Depression (CES-D) was used to measure depressive symptoms [44]. Participants were inquired about the frequency of different symptoms during the last week in CHARLS 2015. With these 10 items, the CES-D-10 can be used to diagnose depressive disorder and its severity and has been proved to be validated and reliable for the Chinese elderly [45]. Responses ranged from 0 = less than one day, 1 = one to two days, 2 = three to four days, 3 = five to seven days, and were summed to create a total score ranging from 0 to 30. A higher score of CES-D-10 indicates increased depressive symptoms. The scale of CES-D-10 was found to be reliable as the Cronbach’s alpha (α) for the present sample was 0.791. In this study, depressive symptoms were measured by the score of CES-D-10, and used as a continuous variable.

#### 2.2.2. Labor Force Participation (LFP)

Labor force participation (LFP) was measured by working hours annually. Lacking of stable work, rural seniors always engage in various productive or paid work [15]. Thus, the summed hours of agricultural work, non-farm self-employed work, and working for others were used to measure LFP among rural older adults. In the questionnaire, three questions were asked in each work, as follows: (a). How many months did you engage in agricultural work, non-farm self-employed work, and working for others in the past year? (b). How many days a week did you work on average in the past year? (c). How many hours did you work per day on average in the past year? The sum of working hours annually was created by the above questions. The LFP and Ln (LFP + 1) were used as continuous variables in this study.

#### 2.2.3. Pension Income

In 2009, the New Rural Pension Scheme (NRPS) was launched in China as a formal arrangement for rural old age support. Over 400 million rural residents had contributed to the NRPS, and at least 100 million rural elderly had become pension beneficiaries by the end of 2014 [11,46]. NRPS consists of two parts, one is noncontributory basic pension (BP) and the other is a voluntary funded defined contribution (FDC) component. Pension payments will be provided to rural residents when they reach the age of 60. BP is rolled out at the provincial level, and entirely financed by the central government in central and western regions [47]. On the contrary, in the affluent eastern provinces, it is half financed by the central government, and half by local governments. At the beginning, all rural residents aged 60 and over can receive BP even with no contribution to FDC. The only condition of receiving BP is that their adult children contribute to FDC. In 2015, the BP was at least 70 CNY (around 10 USD) per month, while some rich provinces provided a much higher amount (e.g., BP of Beijing is 705 CNY and BP of Shanghai is 660 CNY). The FDC component of NRPS is rolled out at the county level, and consists of individual’s payment and local government subsidy. Rural residents can choose the payment grades, which include basic fixed grades (100, 200, 300, 400, 500, 600, 700, 800, 900, 1000, 1500, and 2000 CNY per year) [48] on a voluntary basis as well as flexible ones introduced by local governments. The local government subsidy is prescribed no less than 30 CNY per month, and will increase as the amount of individual’s payment increases. In the present study, we treated the variables PI and Ln (PI + 1) as continuous variables.

#### 2.2.4. Covariates

Sociodemographic characteristics in this study include age, gender, education years, marital status, and functional limitations. In CHARLS 2015, education level was classified into 12 categories, ranging from 1 (illiterate) to 12 (doctoral degree). We converted education level into education years. Marital status was dummy coded “1” as “have a life partner”, “2” as “have no spouse”. Functional limitations were measured by the sum of nine items, which assessed the difficulty with jogging, walking, getting up from a chair after sitting for a long period, climbing, stooping, kneeling, or crouching, extending arms, lifting, and picking up a coin. The summed scores of functional limitations ranged from 0 to 27. All covariates were used as continuous variables except for marital status.

### 2.3. Analytical Strategy

**Figure 2:**
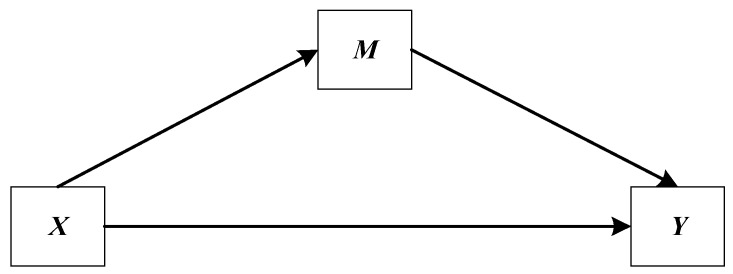
A simple mediation model (Model 4) in the path diagram form.

**Figure 3:**
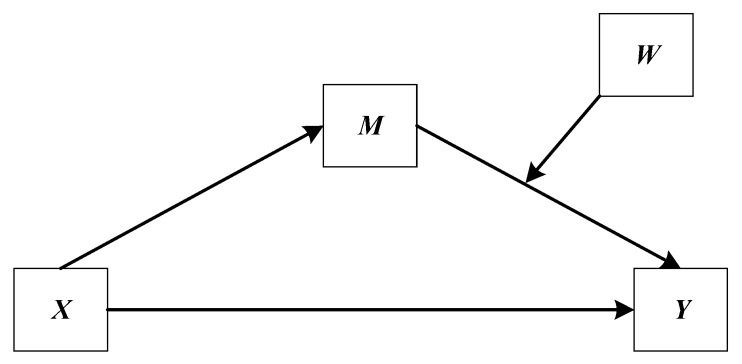
A moderated mediation model (model 14) in the path diagram form.

The data was analyzed using IBM SPSS Statistics for Windows version 20.0 (IBM Corp., Armonk, NY, USA) in three stages. The first stage was preliminary analysis. Chi-square test (for categorical variable) and Mann–Whitney U test (a nonparametric test for continuous variables) were carried out at this stage to examine differences in study variables by gender. Due to the skewed distributions of the study continuous variables, the standard parametric significance test would not be very exact, thus the Mann–Whitney U test was conducted. In the second stage, a simple mediation model (model 4) of the PROCESS Macro version 3.3 by Andrew F. Hayes for SPSS [49] was performed to examine the direct relationship between PI and depressive symptoms, as well as the indirect effect of LFP on this relationship. This model reflects a causal sequence in which X affects Y indirectly through mediator variable M, and can be used to test Hypothesis 1 and 2 in this study (see in Figure 2). In this model, X is postulated to affect M, and this effect then propagates causally to Y. This indirect effect represents the mechanism by which X transmits its effect on Y. According to this model, X can also affect Y directly. In other words, a direct effect of X is independent of X’s influence on M [50]. In the third stage, a moderated mediation model (model 14) of the PROCESS Macro was performed to examine the moderation effect of gender on the link between LFP and depressive symptoms. This model allows the effect of X on M in a mediation model to be moderated by W, and can be used to test Hypothesis 3 in our study (see in Figure 3). In this model, M is estimated as a linear function of X, and Y estimated as a linear function of both M and X, with the effect of M on Y modeled as linearly related to W. In order to reduce the differences in variable values and the influence of the outliers on the estimates of regression models, PI (Ln + 1) and LFP (Ln + 1) were used in the regression analyses.

Furthermore, we preferred the bootstrapping method to produce confidence intervals (CIs). Bootstrapping is a popular resampling strategy for estimation and hypothesis testing, and does not make the unwarranted assumption of normality of the sampling distribution of the relative indirect effect [51,52]. When we never compute a standard error but base our inferences on the number of bootstrapping samples yielding statistics in various ranges, it requires a larger value of the samples, at least 1000 [53]. The ideal number is 5000 or more [50]. In this study, 5000 bootstrapping samples were sufficient, which led to extremely consistent results using 10,000 samples. If CI includes zero, we cannot exclude no relationship between the moderator and the indirect effect from the realm of plausibility. However, if CI does not include zero, this leads to the inference that the size of the relationship between the indirect effect and the moderator is not zero.

## 3. Results

### 3.1. Preliminary Analysis

The mean values of the variables of interest for the total sample, the male and female subsamples are presented respectively in Table 1. Among the 2709 participants, 56.1% were women, and 78.7% had a life partner. The mean age, education years, and score of functional limitations stood at 68.3 years, 3 years, and 6.9 points respectively. Averagely speaking, the logged PI and LFP was 4.4 and 3.7, which can be converted to 104.9 RMB per month and 779 h per year accordingly. Lastly, the average score of depressive symptoms was 10.2 points. In terms of contrast statistics, there were substantial differences between the older male and older female subsamples with respect to age, marital status, education years, functional limitations, LFP, and depressive symptoms, except for PI and Ln (PI + 1). Furthermore, results indicated that men participants were more likely to be older (Cohen’s d = 0.127, *p* < 0.05), have a life partner (Cramer’s V = 0.122, *p* < 0.001), be educated longer (Cohen’s d = 0.768, *p* < 0.001), have lower functional limitations (Cohen’s d = −0.095, *p* < 0.01), higher LFP (Cohen’s d = 0.293, *p* < 0.01), and experience milder depressive symptoms (Cohen’s d = −0.322, *p* < 0.001). Differences between the two subsamples approximated small to medium effects with respect to marital status, LFP, Ln (LFP + 1) and depressive symptoms. Difference about education years was meaningfully significant with a medium to large effect size when comparing the older male with the older female.

### 3.2. The Results of Mediation Estimates

PROCESS macro (Model 4) was used to test for the direct and indirect effects in the overall sample. The results of mediation estimates are shown in Table 2. In Hypothesis 1, PI was expected to have a direct effect on depressive symptoms among the rural elderly. When controlling for age, education years, marital status, and functional limitations, the results of mediation estimates revealed that PI significantly correlated with depressive symptoms (β = −0.098, *p* < 0.001). Moreover, an increase in PI was directly associated with a decrease in depressive symptoms. Thus, Hypothesis 1 was supported. In Hypothesis 2, PI was assumed to have an indirect effect on depressive symptoms among the participants through LFP. The results of mediation estimates indicated that there was a significantly negative relationship between PI and LFP (β = −0.047, *p* < 0.01). However, the association between LFP and depressive symptoms was not significant (β = 0.035, *p* = 0.0707). The 95% CI for unstandardized indirect effect of PI on depressive symptoms included zero and some positive values indicating that the indirect effect of PI on depressive symptoms among the overall participants through LFP was not significant (effect = −0.0216, 95% CI = [−0.0580, 0.0023]). Thus, Hypothesis 2 was not supported. 

### 3.3. The Results of Moderated Mediation Estimates

**Figure 4:**
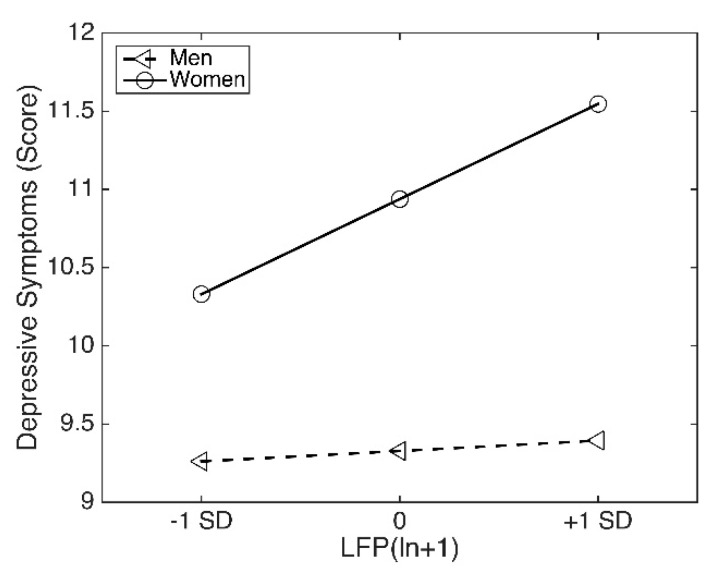
Conditional effect of gender on the association between LFP and depressive symptoms.

As noted, Hypothesis 3 predicted that gender would moderate the indirect association between PI and depressive symptoms through LFP. In order to estimate the moderated indirect effects, a conditional process model (model 14) was applied. The results of moderated mediation estimates are shown in Table 3. When controlling for age, education years, marital status, and functional limitations, the links from PI to LFP (β = −0.047, *p* < 0.01) and from LFP to depressive symptoms (β = 0.054, *p* < 0.01) were significant. Furthermore, the interaction between LFP and gender was statistically significant (β = 0.039, *p* < 0.05), indicating that gender was moderating the mediation of the effect of PI on depressive symptoms by LFP. The index of moderated mediation reflects the difference between conditional indirect effects. In this study, 95% CI for the index of moderated mediation through gender did not include zero (index = −0.0490, 95% CI = [−0.1210, −0.0009]). Accordingly, we could claim that any two conditional indirect effects estimated at different values of gender were statistically different from each other, and thus moderation of the indirect effect mentioned above by gender was plausible. Specifically, for older females, 95% CI for the conditional indirect effect of PI on depressive symptoms via LFP did not include zero (effect = −0.0060, 95% CI [−0.0456, −0.0311]), which indicated this indirect effect was plausible only in the condition of the female subsample (effect = −0.0550, 95% CI [−0.1141, −0.0092]). A simple slope analysis was applied to further investigate the moderating role of gender in the association between LFP and depressive symptoms (see in Figure 4). As predicted, the relationship between LFP and depressive symptoms was significantly stronger among the older women than their counterparts. Thus, Hypothesis 3 was supported. 

## 4. Discussion

This study examined the direct and indirect relationships between PI, LFP, geriatric depressive symptoms, and gender in rural China. This study contributes to the literature in the following ways. Consistent with previous studies, our study points out that older women suffered from a higher level of depressive symptoms (according to CES-D 10) than their men counterparts [55]. An investigation on post-stroke depression (PSD; according to the Hamilton depression rating scale (HDRS)) in China also found that female subjects were more likely to develop PSD than their male counterparts [56]. One epidemiological study further confirmed higher prevalence of depressive disorders and significant differences of the clinical presentation of depressive symptoms in females in relation to males [57]. One explanation for these differences is that women exhibit a specific mood disorder associated with physiological characteristics. The other important cause may be a lower socioeconomic status (SES) of women especially in developing regions [58,59]. As a result, the scores of depressive symptoms are commonly even higher among older women. In addition, our findings are consistent with previous studies in which income exhibits significant association with depressive symptoms [60]. Although results from previous studies suggest a similar conclusion that an increase in income may reduce depressive symptoms, much of the studies are carried out in developed countries [16,17]. Moreover, the literature on cash transfer programs and older population in developing regions are limited when considering the problem of depression. Our study supplements the existing literature by examining these associations among geriatric population in rustic China. Additionally, corresponding to previous research in China [61], NRPS payment was suggested to have protective effect on mental health in our study. The possible explanation is that PI may account for a larger share of income for low-income population, thus, directly generates much more utility of mental well-being [21].

Secondly, this study discovered a significantly negative relationship between the income from NRPS and LFP in late life. In this way, our finding is in line with some previous research, which demonstrates a negative link between PI and LFP with two possible explanations [35]. One is that the income from NRPS provides the rural elderly with financial incentives to retire [34]. Thus, they are not obliged to engage in physically strenuous labor any more. The other is that the income from NRPS increases the likelihood that individuals consider themselves pensioners with negative attitudes to ageing, and reduce labor supply [35]. In addition, this study found no evidence that LFP had a connection with depressive symptoms among overall study population. In other words, LFP was not a significant mediator between PI and depressive symptoms when studying the overall older population. To our knowledge, this study is the first to explore the missing link between PI and depressive symptoms through an investigation of LFP among the older adults in rural China. In order to explore the role of LFP in depth, future studies might expand on this topic by introducing other variables (e.g., LFP willingness and informal LFP).

Thirdly, the main objective of this research was to examine the moderated mediation effects of LFP and gender. According to the results of moderated mediation estimates, gender significantly correlated with depressive symptoms, and moderated the link between LFP and depressive symptoms in the study population. Specifically, this link was salient for older women, rather than men. In rural China, gender norms about the superiority of men and the division of labor across gender are prevailing features of society. According to the traditional gender roles, family leadership and breadwinning are ascribed to men, caregiving for families is ascribed to women [62,63]. Both older men and older women have to participate in labor force market especially when PI decreases, which could engender a sacrifice of their social interests. In addition, activities and social interests are suggested to be linked to a reduced level of depressive symptoms in late adulthood [64], especially for the older women who are more interested in social activities [65]. At the same time, participating in paid and productive work may lead to a sense of role conflict associated with depressive symptoms among older women rather than older men. Even though this study elucidates that due to significant gender difference, older men have a higher LFP rate than older women [37], there is no evidence to suggest that LFP is correlated with depressive symptoms among the rural elderly in China. In other words, the pathway that PI affects depressive symptoms in older men is unclear. In understanding this conundrum, it is evident that more research is warranted.

The strength of the current study is that we focus on the potential pathway from PI to depressive symptoms, using a large national sample of rural older adults. To the best of our knowledge, our findings supplement the existing literature by examining the association between income from NRPS and depressive symptoms for older men and women separately. This study, however, still had several limitations worth noting. First, depressive symptoms were measured using a short-come of CES-D-10 scale, and analyzed as a continuous variable. More accurate scales (e.g., Hamilton depression rating scale and Montgomery and Asberg depression rating scale) and validated cutoffs need to be established for Chinese elderly in the future. Second, we excluded surveys with missing values, which may not represent a random subset of the larger sample. Thereby, precision of estimates was reduced. Third, LFP was measured by working hours for breadwinning, excluding the domestic work in which women always engage. Since there was significant gender difference in LFP according to the traditional labor roles, this measurement might lead to the result that the rate of LFP in women participants was significantly lower than their counterparts. The future work is to further explore different modes of LFP, and investigate other mediators between PI and depressive symptoms, particularly in men. Lastly, this study was based on a cross-sectional dataset, thus, no causal inferences were reported.

## 5. Conclusions

Geriatric depression in rural China is a common mental illness, which can be relevant to poor welfare and unceasing work. This study extends previous findings by demonstrating the pathway from public pension to depressive symptoms through LFP across gender in rural China. In conclusion, the results are consistent with recent studies indicating that an increase in the income from NRPS is related to a decrease in LFP and depressive symptoms. In addition, LFP can act as a mediator in the relationship between PI and depressive symptoms in older female subsample. These results suggest that public pension can potentially have a gender-based impact on mental health among the vast majority of the rural older population, and significantly contribute to the intervention of depression in the older female. Our findings may be useful for policy makers and mental health therapists to better understand the outcomes of NRPS and predictors of depressive symptoms. 

## Figures and Tables

**Table 1:** Description of analytic variables.

Variables	Total (*N* = 2709)		Men (*n* = 1189)		Women (*n* = 1520)		Men vs. Women
	Mean	SD	Mean	SD	Mean	SD	ES
Age	68.3	6.1	68.7	6.2	68.0	6.0	0.127
Marital Status							0.122
Have a life partner (*N*, %)	2132	78.7	1003	84.4	1129	74.3	
Have no spouse (*N*, %)	577	21.3	186	15.6	391	25.7	
Education Years	3.0	3.3	4.3	3.3	2.0	2.8	0.768
Functional Limitations	6.9	1.9	6.8	1.8	7.0	2.0	−0.095
PI (Ln + 1)	4.4	0.5	4.4	0.5	4.4	0.5	−0.022 ^ns^
PI (RMB)	104.9	172.7	105.5	188.8	104.4	159.0	0.006 ^ns^
LFP (Ln + 1)	3.7	3.5	4.3	3.4	3.3	3.4	0.312
LFP (Hours)	779.0	1159.5	967.9	1277.3	631.3	1035.0	0.293
Depressive Symptoms	10.2	6.9	9.0	6.3	11.2	7.1	−0.322

Note: PI—Pension Income; PI (Ln + 1)—Ln (PI + 1); LFP—Labor Force Participation; LFP (Ln + 1)—Ln (LFP + 1). The results of the Chi-square test (for the categorical variable) and Mann–Whitney U test (for continuous variables) were significant (*p* < 0.05) except those with superscript ^ns^. ES = effect size estimates (Cramer’s V for categorical variable and Cohen’s d for continuous variables). The interpretations of effect sizes were built on the values: small effect size (Cramer’s V = 0.1/(q − 1)^0.5^, Cohen’s d = 0.2), medium effect size (Cramer’s V = 0.3/(q − 1)^0.5^, Cohen’s d = 0.5) and large effect size (Cramer’s V = 0.5/(q−1)^0.5^, Cohen’s d = 0.8), q is the minimum value between the number of rows and columns [54].

**Table 2:** The results of mediation estimates.

Variables	Unstandardized Coefficients	SE	Standardized Coefficients (β)		t	*p*	R^2^
Outcome: LFP (ln + 1)							
Constant	17.52	0.89			19.76	0.0000	0.1237
PI (Ln + 1)	−0.31	0.12	−0.047		−2.59	0.0098	
Outcome: Depressive Symptoms							
Constant	13.62	1.88			7.23	0.0000	0.1252
PI (Ln + 1)	−1.30	0.24	−0.098		−5.41	0.0000	
LFP (Ln + 1)	0.07	0.04	0.035		1.81	0.0707	
Unstandardized Indirect Effect		BootSE		LLCI		ULCI	
−0.0216		0.0157		−0.0580		0.0023	

Note: PI (Ln + 1)—Ln (PI + 1); LFP—Labor Force Participation; LFP (Ln + 1)—Ln (LFP + 1); LLCI—lower 95% level confidence interval; ULCI—upper 95% level confidence interval.

**Table 3:** The results of moderated mediation estimates.

Variables	Unstandardized Coefficients	SE	Standardized Coefficients (β)	t	*p*	R^2^
Outcome: LFP (ln + 1)						
Constant	13.80	0.89		15.56	0.0000	0.1237
PI (Ln + 1)	−0.31	0.12	−0.047	−2.59	0.0098	
Outcome: Depressive Symptoms						
Constant	12.56	1.84		6.82	0.0000	0.1381
PI (Ln + 1)	−1.34	0.24	−0.101	−5.66	0.0000	
LFP (Ln + 1)	0.11	0.04	0.054	2.79	0.0053	
Gender	1.61	0.27	0.117	5.92	0.0000	
LFP (Ln + 1) *Gender	0.16	0.07	0.039	2.17	0.0304	
Index of Moderated Mediation (Difference between conditional indirect effects)			Index	BootSE	LLCI	ULCI
			−0.0490	0.0312	−0.1210	−0.0009
Conditional Indirect Effects		Gender	Effect	BootSE	LLCI	ULCI
PI—LFP—Depressive Symptoms		Men	−0.0060	0.0183	−0.0456	0.0311
		Women	−0.0550	0.0276	−0.1141	−0.0092

Note: PI (Ln + 1)—Ln (PI + 1); LFP—Labor Force Participation; LFP (Ln + 1)—Ln (LFP + 1); LLCI—lower 95% level confidence interval; ULCI—upper 95% level confidence interval. The values of moderated mediation index and conditional indirect effects were unstandardized.
